# Adaptive Evolution of HIV at HLA Epitopes Is Associated with Ethnicity in Canada

**DOI:** 10.1371/journal.pone.0036933

**Published:** 2012-05-31

**Authors:** Manon Ragonnet-Cronin, Stéphane Aris-Brosou, Isabelle Joanisse, Harriet Merks, Dominic Vallee, Kyna Caminiti, Paul Sandstrom, James Brooks

**Affiliations:** 1 National HIV and Retrovirology Laboratories, Public Health Agency of Canada, Ottawa, Canada; 2 Department of Biology, University of Ottawa, Ottawa, Canada; 3 Department of Medicine, University of Ottawa, Ottawa, Canada; British Columbia Centre for Excellence in HIV/AIDS, Canada

## Abstract

Host immune selection pressure influences the development of mutations that allow for HIV escape. Mutation patterns induced in HIV by the human leukocyte antigen (HLA) are HLA-allele specific. As ethnic groups have distinct and characteristic HLA allele frequencies, we can expect divergent viral evolution within ethnicities. Here, we have sequenced and analyzed the HIV *pol* gene from 1248 subtype B infected, treatment-naïve individuals in Canada. Phylogenetic analysis showed no separation between *pol* sequences from five self-identified ethnic groups, yet fixation index (*F_ST_*) values showed significant divergence between ethnicities. A total of 17 amino acid sites showed an ethnic-specific fixation pattern (0.015<*F_ST_* <0.060, *p*<0.01), and 27 codons were inferred to be under positive selection (*p*<0.01), with each set of sites strongly associated with HLA sites (*p* = 1.78×10^−6^ and *p* = 1.91×10^−7^, respectively). Within the *pol* gene, eight sites under HLA selective pressure were correlated with ethnicity, indicating ‘adaptive divergence’ between the groups studied. Our findings highlight challenges in HIV vaccine design in ethnically diverse countries with subtype B epidemics.

## Introduction

Immune-mediated selection pressure is one of the strongest forces driving HIV evolution, with human leukocyte antigen (HLA) proteins playing a critical role. HLA cell-surface proteins bind and display antigenic epitopes cleaved from viral proteins. Epitope display initiates the cytotoxic T lymphocyte (CTL) response and the destruction of HIV infected cells. However, HLA proteins are able to display only epitopes to which they bind tightly. Consequently, amino acid substitutions within HIV epitopes can interfere with the CTL response by decreasing the efficiency of epitope binding, disrupting the intracellular processing of epitopes or impairing recognition by T cells. Thus, the incredibly high mutation rate of HIV [1], combined with strong selective pressure, facilitate immune escape through the mutation of sequences that are targeted by the CTL response [2]–[4].

HLA alleles are extremely diverse, with each allele capable of binding different, but overlapping, sets of viral epitopes. Among populations which share common HLA alleles, HIV can evolve in parallel at HLA-associated sites due to the fixation of HLA-allele specific escape mutations [5]–[7]. Concordantly, the frequency of HLA-associated polymorphisms in circulating HIV isolates has been shown to reflect the prevalence of HLA alleles in different populations [8]. Links between HLA alleles and the HIV mutation patterns they generate have been established by multiple large scale association studies on cohorts for which both HLA allele data and viral sequences are available [5], [6], [9]–[12]. In the largest study of its type, Brumme *et al.* mapped polymorphisms due to HLA immune escape across HIV genome sequences within a multi-center cohort of over 1500 HIV patients (International HIV Adaptation Collaborative, or IHAC) from the USA, Canada and Australia [6]. In a subsequent study, John *et al.* demonstrated that at an 8–11 mer resolution, HLA responses differed according to ethnicity, establishing that there were distinct inheritable patterns of HIV immune response [7]. In a diverse population in the USA, ethnic-specific selection patterns were observed in HIV because frequencies of HLA alleles resolved at a high level differed across the groups studied. Congruently, Kosakovsky Pond *et al.* found that the strength of selection varied at sites in HIV between two genetically distinct populations [13].

Similar to the USA, the Canadian HIV epidemic is ethnically heterogeneous. According to surveillance data reported in 2008 and for which ethnicity data was available, 44.3% of HIV cases were Caucasian, 33.3% Aboriginal, 11.6% African-Caribbean, 4.5% Asian, and 4.1% Latin-American [14]. Of particular note is the over-representation of Aboriginals in the Canadian HIV epidemic, estimated to account for 8% of prevalent infections [15] but only 4% of the population [16]. Population studies in the USA have shown that HLA allele frequencies differ significantly between the five major ‘outbred’ ethnic groups: African-Caribbean, Asians, Caucasians, Native Americans and Latin-Americans [7], [17]. To gain insight into the forces driving the evolution of the HIV epidemic, we sought to investigate whether HIV sequences coming from different ethnic groups in Canada exhibited characteristic mutation patterns resulting from shared host-driven selective pressures.

Since HLA allele frequency data are currently not available for association studies in the Canadian population we studied, we used a recently developed method to compare host selection pressure between populations in the absence of HLA allele frequency data [18]. In order to examine the differences in selective pressure within different ethnic groups, we compared site-specific frequencies of amino acids in HIV *pol* sequences classified according to ethnicity. This method offers the additional advantage of not requiring phylogenetic separation of sequences for the populations studied [18]. We found divergent HIV sequence patterns among ethnic groups at 8 sites under positive selection that have been shown to mutate under HLA-associated immune pressure. Results are consistent with differential HIV-1 adaptation to HLA class I alleles among ethnic groups in Canada.

## Results

### Epidemiological Characteristics of the Study Population

Long term infections are most likely to bear evolutionary imprints resulting from the host’s cellular immune response and would therefore be the most relevant to the analysis. In order to maximize the probability that observed mutation patterns were due to HLA selective pressure within the subject under study, and not reflecting immune selection from the transmitting partner, we included only samples from long-term infections (older than 155 days), as determined by the capture enzyme immunoassay or BED-CEIA test [19]. Sequences from 1248 ethnicity-typed subtype B samples, from established infections, were included. Sequences were separated into five ethnic groups previously demonstrated to differ in HLA allele frequencies in North America [17] (Table 1): Caucasian (907, 72.68%), Aboriginal (179, 14.34%), African-Caribbean (23, 1.84%), Asian (81, 6.49%) and Latin-American (58, 4.65%). The 1239 bp (413 amino acids) sequenced *pol* fragment encompasses the entire protease region (PR, 297 bp, 99 aa) and the first 942 nucleotides of the reverse transcriptase gene (RT, 314 aa).

**Table 1:** Epidemiological characteristics of the population.

		Aboriginal	African-Caribbean	Asian	Caucasian	Latin-American	All ethnicities
**Sex**	Female	73	9	6	105	3	**196**
	Male	105	14	75	801	54	**1049**
	Other/unknown	1	0	0	1	1	**3**
**Age**	<20	3	0	0	4	0	**7**
	20–29	33	3	18	129	23	**206**
	30–39	67	12	34	255	17	**385**
	40–49	62	4	16	326	12	**420**
	>50	14	4	13	193	4	**228**
	Other/unknown	0	0	0	0	2	**2**
	Mean	37.39	39.74	37.99	41.57	34.77	**40.40**
**Exposure category**	MSM	21	6	47	448	35	**557**
	MSM/IDU	7	1	1	33	0	**42**
	IDU	95	2	6	226	5	**334**
	HET	46	10	23	172	14	**265**
	Other/unknown	10	4	4	28	4	**92**
**Total**		**179**	**23**	**81**	**907**	**58**	**1248**

**MSM** men who have sex with men, **IDU** intravenous drug users, **HET** heterosexual.

### Viral Divergence Cannot be Explained by Phylogenetic History

**Figure 1:**
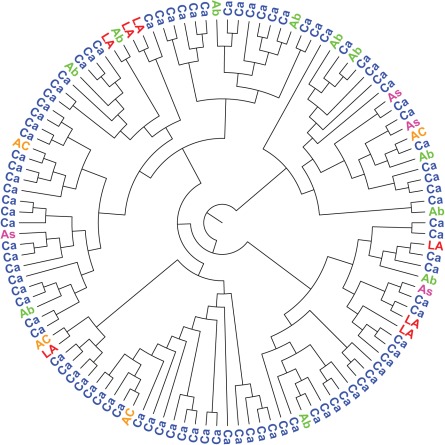
Phylogenetic subtree 2. The phylogenetic relationships between taxa in the subtree were reconstructed in BEAST for subsequent analysis in BaTS. For graphic visualization, a maximum clade credibility tree was generated in TreeAnnotator and annotated in FigTree. Taxa are colored by ethnicity: Aboriginal (Ab, green), African-Caribbean (AC, orange), Asian (As, pink), Caucasian (Ca, blue), Latin-American (LA, red).

Because observed sequence patterns could be due to founder effects and phylogenetic clustering among ethnic groups, it was necessary to identify *a priori* phylogenetic clustering. A maximum likelihood (ML) tree containing 1272 sequences was first constructed with FastTree 2.1 [20] under the most appropriate model selected by jModelTest [21], which was the general time reversible model with among-site rate heterogeneity (GTR+Γ). Two apparent monophyletic clusters associated with common ethnicity were removed, and ML tree reconstruction was repeated on the remaining 1248 sequences. The APE package [22] implemented in R [23] was then used to sample subtrees randomly across the tree for further Bayesian phylogenetic analysis in BEAST [24]. Seven subtrees containing 88–161 sequences were sampled, covering 66.4% of the tree (829/1248 sequences). Subtrees were then tested for clustering of ethnicities in BaTS (Figure 1; see Figure S1 for all other subtrees) [25].

Within each subtree, terminal nodes were annotated with our character of interest (ethnicity) and we tested whether the distribution of ethnicity on the phylogeny was non-random. Association indices and parsimony scores both indicated an absence of phylogenetic clustering by ethnic group in any of the subtrees (Table 2). Therefore, on the final tree, sequences from ethnic groups were no more clustered than expected by chance, and we could proceed with testing whether *pol* sequences showed evidence of ethnic-specific mutation patterns.

**Table 2:** Results from the analysis of seven subtrees in BaTS.

	*n*	AI	*p*	PS	*p*
**Subtree 1**	161	8.43	0.09	50.33	0.25
**Subtree 2**	118	5.06	0.50	24.74	0.32
**Subtree 3**	152	7.70	0.15	44.53	0.12
**Subtree 4**	99	3.92	0.25	21.05	0.07
**Subtree 5**	88	3.72	0.70	15.98	1.00
**Subtree 6**	91	2.52	0.22	12.95	0.08
**Subtree 7**	121	4.71	0.85	22.89	0.36

***n*** is the number of sequences in each subtree. The results of two statistics are shown: **AI** association index and **PS** parsimony score. *p* values >0.05 indicate that sequences are not clustered by ethnicity in the subtrees.

### Seventeen Sites in *Pol* are Divergent Between Ethnicities

**Figure 2:**
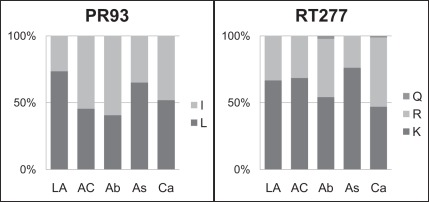
Amino acid frequency distributions at sites PR93 and RT277. For each ethnic group, the frequencies of alternative amino acids are shown for two sites in protease (PR) and reverse transcriptase (RT). Amino acids represented are: leucine (L), isoleucine (I), glutamine (Q), arginine (R) and lysine (K). *F_ST_* values at PR93 and RT277 are 0.06 and 0.0521, respectively. Notation of ethnicities is as follows: Aboriginal (Ab), African-Caribbean (AC), Asian (As), Caucasian (Ca), and Latin American (LA).

In spite of this lack of shared viral ancestry within ethnic groups, an analysis of molecular variance (AMOVA) [26] of 413 amino acid sites in the *pol* region demonstrated significant divergence in amino acid composition between ethnicities at 17 sites (*p*<0.01; Figure S2). Divergent sites were highly polymorphic, as compared to the rest of the *pol* sequence, and their average entropy was seven times higher (0.28 and 0.04; respectively, *p*<5×10^−20^). The extent of population differentiation at these sites was measured through the fixation index (*F_ST_* [27]) that quantifies the proportion of the observed variation that is contained within the subpopulation (‘*S* ’, here, ethnic group) as compared to the total population (‘*T*’). Measured in this way, population differentiation could either be indicative of divergence in amino acid composition between all groups, or could point to a deviation within a single ethnic group. Significant *F_ST_* values (at the 1% level) ranged from 1.5% to 6.0%, signifying that at sites that are divergent between groups, ethnic subgrouping accounted for up to 6% of the observed variation. At all but two of these 17 sites, the most common amino acid was conserved across ethnic groups; only the frequencies of amino acids differed between groups. Patterns of dominance were nevertheless observed at the remaining 15 sites. The highest *F_ST_* values were noted at sites where the most prevalent amino acid differed between ethnicities, PR93 (*F_ST_* = 0.060) and RT277 (*F_ST_* = 0.052, Figure 2; Figures S2,S3).

When the AMOVA was performed on all 1272 sequences (including two clusters of sequences from Aboriginal patients), the same 17 sites were identified, with *F_ST_* values changing only slightly (data not shown).

### Divergent Sites are Strongly Associated with HLA Sites and Sites Under Positive Selection

**Figure 3:**
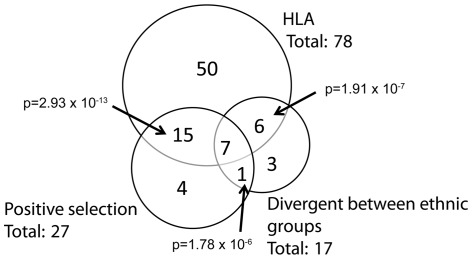
Overlap between sites of interest. Of a total of 413 amino acid sites, considerable overlap is observed between HLA-associated sites, sites under positive selection and sites divergent between ethnicities. Associations were tested using Fisher’s exact test. Note that all three tests take into account the 7 sites in the center of the diagram.

In order to understand the origin of this differentiation at viral sites relative to ethnicity, we further investigated the role of HLA-driven convergent selective pressures. Based on the literature, 78 sites known to be HLA-associated (HLA+) were mapped across the sequenced *pol* region [6]. In parallel, using the Single Ancestor Counting algorithm in HyPhy [28], 27 codons were inferred to be under positive selection (Pos+, *p*<0.01). As most selective pressure in HIV is immune-mediated [12], we expected HLA+ and Pos+ sites to be strongly associated, which was indeed the case (*p* = 2.93×10^−13^, Fisher’s exact test, Figure 3).

Sites divergent between ethnicities were also strongly associated with both HLA+ (*p* = 1.91×10^−7,^ Fisher’s exact test) and Pos+ sites (*p* = 1.78×10^−6^, Fisher’s exact test, Figure 3). Eight codons were both divergent between groups, and inferred to be under positive selection: PR35, PR93, RT135, RT162, RT245, RT277, RT293 and RT297 (Figure S2). According to the definition put forward by Perez-Sweeney *et al.* [18], these sites can be considered putatively adaptive divergent sites, *i.e.* selective pressure may differ at these codons between the groups studied. Of these, all except RT293 were also on the list of HLA-associated sites produced by Brumme *et al.* [6], suggesting that the observed selection pressure is the result of HLA alleles differentially distributed across ethnicities. Moreover, fifteen additional sites were HLA+ and Pos+, although not divergent between ethnicities. These sites appear to be under strong HLA induced selection pressure within the Canadian population, in response to HLA alleles shared among the different ethnic groups examined. Finally, six sites were divergent between groups, and HLA+, but not Pos+ (Figure 3). These sites could either be false positives due to low frequency haplotypes (that is, not divergent), or false negatives (that is, adaptive) not detected by the codon model.

### Strength of Selective Pressure Differs Between Populations Only at a Small Number of Sites

Finally, strength of site-specific selective pressure was directly compared between pairs of populations [13]. A level of significance α = 0.005 (Bonferroni; 5×(5−1)/2 = 10) was chosen to correct for multiple comparisons. The strength of selective pressure differed only at a small number of sites (Table 3). All these sites had previously been detected as divergent by the *F_ST_* test (RT108, RT111), or were included in the list of HLA-associated sites (PR19, RT166, RT198), or both (PR35; Figure S2). Differences in strength of selection pressure on internal branches, indicating substitutions selective at both the individual and population level, occurred only at two sites in protease, PR19 and PR35. Recent adaptations, on branches leading to the tips of the phylogeny, are likely to be maladaptive and purified at the population level, and indeed were not detected by the codon model (Figure S2).

**Table 3:** Differences in strength of selective pressure.

	As	Ab	AC	Ca	LA
**As**					PR19
**Ab**				PR35	
**AC**		RT166			
**Ca**	RT111	PR35, RT108, RT196			
**LA**					

The left hand side of the table (below the diagonal) indicates sites found to differ in strength of selective pressure across the whole tree. The right hand side of the table (above the diagonal) indicates sites found to differ in strength of selective pressure only at internal branches. Analyses compared populations two by two and a level of significance α≤0.005 was necessary for significance. Ethnicities are denoted by two letter codes: Aboriginal (Ab), African-Caribbean (AC), Asian (As), Caucasian (Ca), and Latin-American (LA).

## Discussion

In this study, we characterized HIV sequence diversity and evolution in the ethnically heterogeneous Canadian epidemic in the absence of subject HLA data. We first demonstrated that HIV sequences from patients of different ethnic origins showed distinctive mutation patterns, and that these patterns were not associated with viral ancestry or founder effects. Further analysis confirmed that divergent sites were strongly associated with sites known to be under HLA selective pressure, although there was little evidence for different non-synonymous rates of mutation between pairs of populations. The five ethnic groups studied had previously been demonstrated to have characteristic HLA allele frequencies in different populations [17]; we therefore suggest that HIV is evolving in parallel at these sites within ethnic groups due to shared HLA alleles.

HLA allele frequency data for different ethnic groups is gathered from all published studies and stored in a centralized database [29]. Using this database and known associations between HIV mutations and specific HLA alleles [6], we sought to attribute our observations to HLA alleles more frequently observed in particular ethnic groups. Although HLA data for our study population were not directly available, we used data from comparable ethnic groups in the USA, with Amerindians being used as a proxy for Aboriginals. At most sites, our observations were consistent with available HLA allele frequency data for each ethnic group. For example, at site RT277, the escape mutant R was seen more frequently in Caucasians than in the other ethnic groups. This result is in agreement with a known association between RT277 E and the A*03 allele, found more frequently in Caucasians (up to 30%) than in any other group. Notably, the escape mutant R was the consensus in the IHAC cohort [6], as it would be here, while the susceptible K was more common in Mexico [9]. Broken down by ethnicity, K was the most common amino acid in all groups other than Caucasian. Similarly, at site PR93, we observed high frequencies of B*15 associated escape mutant L in Asians and Latin-Americans. This is consistent with high frequencies of B*15 among Asians (up to 40%). However, we did not find higher frequencies of B*15 among Latin-Americans as compared to other ethnicities in the database. Meanwhile, the high frequency of B*07 escape mutant RT162 C is congruent with the higher frequency of allele B*07 in Caucasians (up to 19%). At sites PR35, RT135, RT245 and RT297, patterns became more complicated to interpret because of high amino acid diversity. Nevertheless, the high frequency of B*57 escape mutant RT245 E among Asians and Latin-Americans is in accordance with the very high frequency of B*57 in these ethnic groups as compared to Caucasians (up to 20% as compared to up to 6% in Caucasians). The degree to which the B*35 escape mutant RT297 A was found among Aboriginals is consistent with the very high frequency of B*35 detected among Amerindians [30] (up to 45% as compared to up to 24% in other ethnic groups). In the context of an association between B*35 and faster disease progression [31], this latter finding is important and is the subject of an ongoing clinical trial in Manitoba (Canadian HIV Trials Network CTNPT 004). Our results are in agreement with reviews highlighting the unique HLA makeup of Amerindian populations, and their restriction in diversity of HLA types [32], [33]. It is therefore very likely that the role of distinct patterns of HLA selection is particularly important in a country like Canada, where a single ethnic group, Aboriginals, is so disproportionately affected by the epidemic.

Although not included in the initial list of HLA sites used, our data showed that RT293 was divergent between ethnicities (*F_ST_* results) and under positive selection when all ethnicities were pooled together. This site has been identified to be under HLA immune pressure in a subsequent HIV/HLA genetic association study performed in Mexico [9]. In that study, the frequency of HLA alleles were distinct as compared to those found in the IHAC cohort and were suggested to reflect the unique Amerindian/Caucasian HLA admixture. Because in our study, amino acid frequencies at this site differed between ethnicities and positive selection was detected, we suggest that selection pressure at this site is indeed HLA-driven in our population, possibly reflecting a similar influence of Aboriginal HLA alleles.

Understanding the forces shaping HIV genetic diversity is crucial to surveillance efforts and for the control of the HIV epidemic. It is clear that heritable factors such as HLA alleles that are associated with ethnicity are strong forces shaping the evolution of HIV [12], [34]. The strength of the method used to characterize HIV evolution here is at least twofold. First, it allows for selection pressures to be compared across groups which do not necessarily phylogenetically separate from each other. Second, the method combines several approaches for detecting differential selection pressure, thus reducing the likelihood of type I and type II errors: (i) we identified putative “adaptively divergent” sites using population genetics and codon-based selection tests; (ii) we evaluated whether these sites corresponded to known HLA epitopes using Fisher’s exact test; (iii) we estimated whether the strength of selective pressure at each site differed between pairs of populations.

Although not entirely discordant, the sites detected by the test to compare selective pressures differed from those detected by the *F_ST_* test. This may be due to a number of reasons. Most importantly, the former test compares only strength of selection between datasets. The model does not allow for variation in nucleotides frequencies at each codon position between pairs of populations. Indeed if different amino acids at a given site were selected for between the two populations, but with the same strength, these would not be highlighted as significant. The *F_ST_* test, on the other hand, detects different patterns of amino acid frequencies between populations but does not incorporate information on strength of selection. In addition, the test comparing selective pressures may be lacking in statistical power as (i) the required correction for multiple comparisons is overly conservative (Bonferroni) and (ii) only a small number of sequences were available, in particular from African-Caribbeans, below the 50 recommended in the HyPhy user manual. Yet sites with the lowest *p* values (but above 0.005) were highly consistent across comparisons (data not shown).

Our results support the conclusion of John *et al*’s that ethnicity data may improve the resolution of HIV/HLA association studies [7]. For statistical power, such studies often group HLA alleles into supertypes. However, within supertypes, the selection effects of different alleles vary significantly. As the distribution of highly resolved HLA alleles is most marked within supertypes, HIV/HLA associations can be masked by this grouping. In our study, different HLA-associated mutation patterns were identified between ethnic groups, even in the absence of HLA data. Thus, as an alternative to classifying HLA alleles to four-digit resolution, we propose that combining 2-digit resolution with ethnicity data may improve the strength of associations demonstrated between HLA alleles and HIV mutations, without prejudicing statistical power. Thorough analysis of cohorts for which HIV sequences as well as ethnicity and HLA allele data are available could be used to evaluate this hypothesis.

Although our findings are compelling, there are several limitations to our analysis. Most importantly, the number of immune-associated polymorphisms would have been greatly increased had HLA allele information been directly available for patients. It is a testament to the strength of HLA-mediated HIV adaptation that it can be detected with such a rough classification as declared ethnicity. The HLA alleles expressed across our ethnic groups are not population-specific. In particular, the Caucasian group is likely to be of admixed origin and to contain subpopulations with different HLA allele frequencies. Moreover, distinct HLA alleles can drive the selection of the same escape mutant [8], potentially obscuring a correlation between the two. In agreement, estimated *F_ST_* values were low, although significant. Nevertheless, despite the potentially imperfect grouping in our analysis, statistical associations were found between mutation patterns and ethnic groups, confirming that our analysis is robust.

Another limitation of this type of analysis is that HLA-associated mutation patterns in HIV may not necessarily reflect the HLA background of the patient currently infected. Although HIV evolves rapidly within a new patient to match his or her HLA type [35], usually during early infection [4], there is also evidence that HIV may revert to wild type [36] or, in the absence of a fitness cost, maintain escape mutants associated with the transmitter’s HLA type. In the acute stage in particular, an adaptation profile specific to the newly infected individual may not yet have been generated. Instead, HLA-associated sites may reflect adaptation to a previous HLA type. To avoid this issue, we used HIV sequences only from patients classified as having established infections according to the BED assay, as over time HIV is more likely to adapt to the new patient’s HLA type.

The dataset under study was generated though HIV surveillance for new diagnoses and presents certain limitations due to small subsets of data. The African-Caribbean population in particular, is greatly under-represented, reflecting the imbalance of subtype B infected ethnic groups in the epidemic. In Canada, the vast majority of HIV cases are among Caucasians and Aboriginals. Nevertheless, we felt that an inclusion of all major ethnic groups, despite their small numbers, would benefit the analysis, in so far as results are more widely applicable to similar ethnically-diverse western epidemics.

The data could also be biased by the unequal distribution of exposure categories across ethnic groups. Specifically, there is an over-representation of intravenous drug users and an under-representation of men who have sex with men among Aboriginals. Polymorphisms transmitted within single exposure category and thus single-ethnicity transmission clusters could artefactually appear ethnic-specific. While in some epidemics sequences cluster significantly by exposure category, overlap between exposure categories is seen in Canadian clusters [37] and so we do not expect this to be the case. There is otherwise a mix of exposure categories across the ethnic groups, and we took care to remove two clusters which may have introduced bias.

We have shown that HLA-associated mutation patterns differ across the ethnicities studied. These observations support ongoing research investigating whether differences in HLA allele frequencies could explain the susceptibility of Aboriginals to HIV. Furthermore, our study suggests that incorporating ethnicity information into future HLA association studies in Canada may be important. Finally, an understanding of ethnic specific host influence on viral evolution is critical for vaccine development, as an effective vaccine will require targeting regions that are reactive across groups.

## Materials and Methods

### Ethics Statement

The Canadian HIV Strain and Drug Resistance Surveillance Program (SDR) receives serum samples from all newly diagnosed, treatment-naïve HIV patients in Canada. The SDR program received approval from the Health Canada Research Ethics Board on October 18th, 2007. De-identified specimens are collected as a function of routine public health surveillance and then anonymized prior to further analysis. During sample collection, basic epidemiological data including age, sex, ethnicity and exposure category are recorded. Between 2002 and 2009, 3648 samples were sent to the National HIV and Retrovirology Laboratory in Ottawa.

### Laboratory Analysis

For each sample, viral RNA was extracted and the HIV *pol* region amplified by RT-PCR for sequencing as previously described [37]. *Pol* sequence fragments from different sets of primers were assembled in BioEdit v7.0.9 [38]. Assembled sequences were translated for in-frame alignment to the NCBI HIV-1 subtype B reference genome (accession number: NC_001802) using TranslatorX [39]. Nucleotide sequences were trimmed to identical length (1239 bp) and deposited in GenBank (HM468501-HM468505, HM468508, HM468510, HM468512-HM468514, HM468516, HM468517, HM468520, HM468523-HM468527, HM468530-HM468535, HM468538-HM468540, HM468542-HM468553, HM468557-HM468559, HM468566-HM468571, HM468573, HM468574, HM468576-HM468580, HM468582, HM468583, HM468585-HM468587, HM468589-HM468591, HM468593, HM468595-HM468598, HM468600-HM468602, HM468604, HM468606-HM468608, HM468611, HM468612, HM468614-HM468617, HM468619, HM468621, HM468624, HM468626-HM468629, HM468631, HM468632, HM468634-HM468636, HM468638-HM468640, HM468642-HM468650, HM468652-HM468660, HM468662, HM468665, HM468666, HM468668-HM468671, HM468674-HM468677, HM468679, HM468680, HM468682-HM468689, HM468691-HM468694, HM468696-HM468698, HM468700-HM468705, HM468707, HM468709-HM468711, HM468713-HM468716, HM468718-HM468720, HM468722, HM468725-HM468728, HM468730-HM468736, HM468741-HM468744, HM468747, HM468749, HM468750, HM468752, HM468756, HM468758, HM468760-HM468762, HM468764, HM468765, HM468768-HM468772, HM468774, HM468775, HM468777, HM468779, HM468781-HM468783, HM468785, HM468792, HM468793, HM468795-HM468797, HM468799, HM468801, HM468802, HM468805-HM468809, HM468813-HM468815, HM468817, HM468818, HM468820, HM468825-HM468828, HM468832, HM468834-HM468839, HM468841, HM468846-HM468848, HM468850, HM468852, HM468853, HM468855-HM468863, HM468866-HM468868, HM468872, HM468874, HM468875, HM468880, HM468883-HM468887, HM468889, HM468892, HM468894, HM468896, HM468897, HM468901, HM468902, HM468908, HM468910-HM468914, HM468917-HM468920, HM468922, HM468925, HM468927, HM468928, HM468931-HM468935, HM468939, HM468941, HM468943, HM468944, HM468948-HM468957, HM468959, HM468964, HM468966, HM468967, HM468970, HM468971, HM468974, HM468978, HM468981, HM468987, HM468994-HM468996, HM469001, HM469003, HM469006, HM469007, HM469009, HM469010, HM469014, HM469015, HM469017-HM469020, HM469024-HM469029, HM469031-HM469033, HM469036, HM469038, HM469040, HM469044, HM469045, HM469047-HM469051, HM469053, HM469055, HM469057, HM469058, HM469061, HM469062, HM469070, HM469073, HM469074, HM469076, HM469080, HM469086-HM469088, HM469090, HM469092, HM469096, HM469098, HM469099, HM469100, HM469102, HM469103, HM469105, HM469110, HM469113, HM469119, HM469121, HM469125, HM469126, HM469128, HM469129, HM469137-HM469139, HM469141, HM469143, HM469144, HM469148, HM469150, HM469151, HM469153, HM469154, HM469161, HM469162, HM469168, HM469170, HM469171, HM469173, HM469174, HM469179, HM469182, HM469183, HM469185, HM469187, HM469188, HM469190, HM469191, HM469201, HM469202, HM469204, HM469207, HM469210, HM469213, HM469215, HM469217, HM469220-HM469223, HM469225, HM469226, HM469228, HM469232, HM469234, HM469237, HM469240, HM469242, HM469246, HM469249, HM469256, HM469273, HM469276, HM469283, HM469286, HM469288, HM469289, HM469292, HM469294, HM469296-HM469299, HM469300, HM469305, HM469306, HM469309, HM469310, HM469312-HM469315, HM469319, HM469320, HM469327, HM469329, HM469331, HM469333, HM469337, HM469338, HM469340, HM469346-HM469348, HM469351, HM469355, HM469356, HM469359, HM469362, HM469364-HM469366, JQ674950-JQ674959, JQ674961-JQ674963, JQ674965-JQ674992, JQ674994-JQ675008, JQ675011-JQ675067, JQ675069-JQ675080, JQ675082-JQ675088, JQ675090-JQ675096, JQ675098, JQ675099, JQ675101, JQ675102, JQ675104-JQ675109, JQ675111-JQ675115, JQ675117-JQ675143, JQ675145-JQ675158, JQ675160-JQ675169, JQ675171, JQ675173-JQ675176, JQ675179-JQ675183, JQ675185-JQ675212, JQ675214-JQ675232, JQ675234-JQ675240, JQ675242-JQ675248, JQ675250, JQ675253-JQ675258, JQ675260, JQ675262, JQ675263, JQ675266, JQ675268, JQ675269, JQ675272, JQ675275, JQ675277-JQ675281, JQ675283-JQ675285, JQ675287, JQ675288, JX014632-JX015114). All sequence manipulations, such as nucleotide to amino acid translations, were carried out in BioEdit. For each sequence, the subtype was determined by submission of *pol* to the REGA HIV-1 Subtyping Tool v2.0 (http://dbpartners.stanford.edu/RegaSubtyping/). In addition, stage of infection was determined for each sample as < or >155 days (recent vs. established, respectively) using the Calypte BED-CEIA™ (capture enzyme immunoassay). The BED-CEIA (or BED for short) measures the proportion of IgG antibodies which are HIV-specific in a sample [19]. Only subtype B samples, which account for the majority of infections circulating in Canada [14], and originating from established infections, as determined by BED, were included. Sequences were divided into five datasets based on ethnicity: Caucasian, Aboriginal, African-Caribbean, Latin-American and Asian, for analysis. HLA allele frequencies have previously been demonstrated to differ between these groups in North America [17].

### Phylogenetic Analysis and Character Association

Phylogenetic interrelationships between 1272 sequences were reconstructed in FastTree 2.1 under a GTR+Γ model, as selected by jModelTest. Two clusters of sequences for which there was an association between ethnicity and phylogeny were removed from the dataset and the ML reconstruction was repeated on 1248 sequences. Using the APE package in R, subtrees were randomly selected for Bayesian phylogenetic analysis in BEAST. Sequence subsets were run in duplicate under a Bayesian Skyline Plot model with 10 breakpoints and linear splines. Convergence was assessed in Tracer after 100 million generations. After elimination of a burn-in period (10–20% of run), a posterior distribution of trees was generated for analysis in BaTS. Terminal nodes were annotated with our character of interest, ethnicity, and the non-random distribution of ethnicity was tested in each subtree.

### Positive Selection and Population Divergence

Sites previously identified to be under HLA-mediated selection [6] were mapped along sequences in the alignment. Sites inferred to be under positive selection (*p*<0.01) were determined using HyPhy in each ethnic group dataset. In datasets exceeding 50 sequences, the SLAC algorithm was employed [40]. The SLAC algorithm calculates observed numbers of non-synonymous (N) and synonymous (S) mutations at each codon in an alignment, and compares these to expected numbers (E[N] and E[S]) in order to estimate selection pressure (dN = N/E[N], dS = S/E[S]). A low dN/dS ratio (<1) indicates purifying selection, while a high dN/dS (>1) suggests diversifying positive selection pressure. In datasets <50 sequences, both the FEL and REL algorithms were used, and only sites appearing in both lists were considered to be under positive selection (as recommended by the HyPhy user manual).

Site-specific entropy values for each amino acid within the alignment were calculated using the Los Alamos Entropy Tool (http://www.hiv.lanl.gov/content/sequence/ENTROPY/entropy_one.html). Entropy is a measure of the variability at each position in an alignment; a site with high entropy is highly variable.

Next, population divergence between ethnic groups was measured at the amino acid level by calculating the Fixation Index (*F_ST_*) using an analysis of molecular variance (AMOVA), as implemented in Arlequin v3.5.1.2 [41]. In order to calculate *F_ST_* between amino acid sequences, positional amino acid frequency data were generated in BioEdit and transformed into a format readable by Arlequin using an in-house Perl script (available upon request).

In order to determine whether sites that were divergent between ethnic groups were associated with sites under positive selection and HLA-associated sites, we used Fisher’s exact test. All statistical analyses were carried out in SPSS [42].

Finally, we estimated whether the strength of selective pressure at each site differed between pairs of populations using two tests [13] implemented in HyPhy. The former compares selection pressure across two trees constructed for populations separately. However, recent non-synonymous changes (on external branches, leading to tips) reflect only adaptation at the host level and may be maladaptive at the population level. Hence the second test discriminates between substitutions occurring on internal branches from those occurring on external branches of the tree.

## Supporting Information

Figure S1 **All the subtrees.** Maximum clade credibility reconstructions for all six remaining subtrees (1; 3 to 7, in order, A to F) evaluated in BaTS. Taxa are colored by ethnicity: Aboriginal (Ab, green), African-Caribbean (AC, orange), Asian (As, pink), Caucasian (Ca, blue), Latin-American (LA, red).
(EPS)

Figure S2 **Sites of interest in the** ***pol*** **sequence.** Only amino acid sites that are HLA-associated (in blue, 78 sites), positively selected (pink, 27 sites) or divergent between ethnicities (green, 17 sites) are shown. The top line indicates position in protease (PR) and reverse transcriptase (RT) in the reference sequence HXB2. The second line is the amino acid in HXB2. For sites divergent between ethnicities, amino acids are displayed in order of frequency. HLA alleles driving selection at each HLA-associated site are displayed in the bottom line.
(EPS)

Figure S3 **Amino acid frequency distributions at sites PR35, RT135, RT162, RT245, RT293 and RT297 (A–F).** For each ethnic group, the frequencies of alternative amino acids are shown for one site in protease (PR) and five sites in reverse transcriptase (RT). Amino acids represented are: alanine (A), cysteine (C), aspartic acid (D), glutamic acid (E), isoleucine (I), lysine (K), arginine (R), serine (S), threonine (T), valine (V), and tyrosine (T). Notation of ethnicities is as follows: Aboriginal (Ab), African-Caribbean (AC), Asian (As), Caucasian (Ca), and Latin American (LA).
(EPS)

## References

[1] Hahn BH, Shaw GM, Taylor ME, Redfield RR, Markham PD (1986). Genetic variation in HTLV-III/LAV over time in patients with AIDS or at risk for AIDS.. Science.

[2] Meier UC, Klenerman P, Griffin P, James W, Koppe B (1995). Cytotoxic T lymphocyte lysis inhibited by viable HIV mutants.. Science.

[3] Phillips RE, Rowland-Jones S, Nixon DF, Gotch FM, Edwards JP (1991). Human immunodeficiency virus genetic variation that can escape cytotoxic T cell recognition.. Nature.

[4] Price DA, Goulder PJ, Klenerman P, Sewell AK, Easterbrook PJ (1997). Positive selection of HIV-1 cytotoxic T lymphocyte escape variants during primary infection.. Proc Natl Acad Sci U S A.

[5] Brumme ZL, Brumme CJ, Heckerman D, Korber BT, Daniels M (2007). Evidence of differential HLA class I-mediated viral evolution in functional and accessory/regulatory genes of HIV-1.. PLoS Pathog.

[6] Brumme ZL, John M, Carlson JM, Brumme CJ, Chan D (2009). HLA-associated immune escape pathways in HIV-1 subtype B Gag, Pol and Nef proteins.. PLoS One.

[7] John M, Heckerman D, James I, Park LP, Carlson JM (2010). Adaptive interactions between HLA and HIV-1: highly divergent selection imposed by HLA class I molecules with common supertype motifs.. J Immunol.

[8] Kawashima Y, Pfafferott K, Frater J, Matthews P, Payne R (2009). Adaptation of HIV-1 to human leukocyte antigen class I. Nature.

[9] Avila-Rios S, Ormsby CE, Carlson JM, Valenzuela-Ponce H, Blanco-Heredia J (2009). Unique features of HLA-mediated HIV evolution in a Mexican cohort: a comparative study.. Retrovirology.

[10] Bhattacharya T, Daniels M, Heckerman D, Foley B, Frahm N (2007). Founder effects in the assessment of HIV polymorphisms and HLA allele associations.. Science.

[11] Carlson JM, Brumme ZL, Rousseau CM, Brumme CJ, Matthews P (2008). Phylogenetic dependency networks: inferring patterns of CTL escape and codon covariation in HIV-1 Gag.. PLoS Comput Biol.

[12] Moore CB, John M, James IR, Christiansen FT, Witt CS (2002). Evidence of HIV-1 adaptation to HLA-restricted immune responses at a population level.. Science.

[13] Kosakovsky Pond SL, Frost SD, Grossman Z, Gravenor MB, Richman DD (2006). Adaptation to different human populations by HIV-1 revealed by codon-based analyses.. PLoS Comput Biol.

[14] Public Health Agency of Canada (2010). HIV and AIDS in Canada..

[15] Public Health Agency Canada (2010). HIV/AIDS Epi Upates - July 2010.

[16] Statscan (2007). Canada Census 2006.

[17] Cao K, Hollenbach J, Shi X, Shi W, Chopek M (2001). Analysis of the frequencies of HLA-A, B, and C alleles and haplotypes in the five major ethnic groups of the United States reveals high levels of diversity in these loci and contrasting distribution patterns in these populations.. Hum Immunol.

[18] Perez-Sweeney B, DeSalle R, Ho JL (2010). An introduction to a novel population genetic approach for HIV characterization.. Infect Genet Evol.

[19] Parekh BS, Kennedy MS, Dobbs T, Pau CP, Byers R (2002). Quantitative detection of increasing HIV type 1 antibodies after seroconversion: a simple assay for detecting recent HIV infection and estimating incidence.. AIDS Res Hum Retroviruses.

[20] Price MN, Dehal PS, Arkin AP (2010). FastTree 2–approximately maximum-likelihood trees for large alignments.. PLoS One.

[21] Posada D (2008). jModelTest: phylogenetic model averaging.. Mol Biol Evol.

[22] Paradis E, Claude J, Strimmer K (2004). APE: Analyses of Phylogenetics and Evolution in R language.. Bioinformatics.

[23] R Development Core Team (2008). R: A language and environment for statistical computing, version Vienna, Austria: R Foundation for Statistical Computing.

[24] Drummond AJ, Rambaut A (2007). BEAST: Bayesian evolutionary analysis by sampling trees.. BMC Evol Biol.

[25] Parker J, Rambaut A, Pybus O (2008). Correlating viral phenotypes with phylogeny: Accounting for phylogenetic uncertainty.. Infect Genet Evol.

[26] Excoffier L, Smouse PE, Quattro JM (1992). Analysis of molecular variance inferred from metric distances among DNA haplotypes: application to human mitochondrial DNA restriction data.. Genetics.

[27] Hartl D, Clark A, Sinauers Associates (2007). Inbreeding, Population Subdivision and Migration.. Principles of Population Genetics.

[28] Kosakovsky Pond SL, Frost SD, Muse SV (2005). HyPhy: hypothesis testing using phylogenies.. Bioinformatics.

[29] Gonzalez-Galarza FF, Christmas S, Middleton D, Jones AR (2011). Allele frequency net: a database and online repository for immune gene frequencies in worldwide populations.. Nucleic Acids Res.

[30] Monsalve MV, Edin G, Devine DV (1998). Analysis of HLA class I and class II in Na-Dene and Amerindian populations from British Columbia, Canada.. Hum Immunol.

[31] Itescu S, Mathur-Wagh U, Skovron ML, Brancato LJ, Marmor M (1992). HLA-B35 is associated with accelerated progression to AIDS.. J Acquir Immune Defic Syndr.

[32] Vina MA, Hollenbach JA, Lyke KE, Sztein MB, Maiers M (2012). Tracking human migrations by the analysis of the distribution of HLA alleles, lineages and haplotypes in closed and open populations.. Philos Trans R Soc Lond B Biol Sci.

[33] Salzano FM (2002). Molecular variability in Amerindians: widespread but uneven information.. An Acad Bras Cienc.

[34] Matthews PC, Leslie AJ, Katzourakis A, Crawford H, Payne R (2009). HLA footprints on human immunodeficiency virus type 1 are associated with interclade polymorphisms and intraclade phylogenetic clustering.. J Virol.

[35] Allen TM, Yu XG, Kalife ET, Reyor LL, Lichterfeld M (2005). De novo generation of escape variant-specific CD8+ T-cell responses following cytotoxic T-lymphocyte escape in chronic human immunodeficiency virus type 1 infection.. J Virol.

[36] Leslie AJ, Pfafferott KJ, Chetty P, Draenert R, Addo MM (2004). HIV evolution: CTL escape mutation and reversion after transmission.. Nat Med.

[37] Ragonnet-Cronin M, Ofner-Agostini M, Merks H, Pilon R, Rekart M (2010). Longitudinal phylogenetic surveillance identifies distinct patterns of cluster dynamics.. J Acquir Immune Defic Syndr.

[38] Hall TA (1999). BioEdit: a user-friendly biological sequence alignment editor and analysis program for Windows 95/98/NT.. Nucleic Acids Symposium Series.

[39] Abascal F, Zardoya R, Telford MJ (2010). TranslatorX: multiple alignment of nucleotide sequences guided by amino acid translations.. Nucleic Acids Res.

[40] Kosakovsky Pond SL, Frost SD (2005). Not so different after all: a comparison of methods for detecting amino acid sites under selection.. Mol Biol Evol.

[41] Excoffier L, Laval G, Schneider S (2005). Arlequin (version 3.0): an integrated software package for population genetics data analysis.. Evol Bioinform Online.

[42] (2009). SPSS for Windows, version 18.0.0 [computer program]..

